# Asarone from Acori Tatarinowii Rhizome prevents oxidative stress-induced cell injury in cultured astrocytes: A signaling triggered by Akt activation

**DOI:** 10.1371/journal.pone.0179077

**Published:** 2017-06-09

**Authors:** Kelly Y. C. Lam, Ping Yao, Huaiyou Wang, Ran Duan, Tina T. X. Dong, Karl W. K. Tsim

**Affiliations:** 1 Division of Life Science, Center for Chinese Medicine, the Hong Kong University of Science and Technology, Clear Water Bay, Hong Kong, China; 2 HKUST Shenzhen Research Institute, Hi-Tech Park, Nanshan, Shenzhen, Guangdong Province, China; University of PECS Medical School, HUNGARY

## Abstract

Acori Tatarinowii Rhizome (ATR; the dried rhizome of *Acori tatarinowii* Schott) is a well-known herb being used for mental disorder in China and Asia. Volatile oil is considered as the active ingredient of ATR, and asarones account for more than 90% of total volatile oil. Here, the protective effects of ATR oil and asarones, both α-asarone and β-asarone, were probed in cultured rat astrocytes. The cyto-protective effect of ATR oil and asarones against tBHP-induced astrocyte injury was revealed, and additionally ATR oil and asarones reduced the tBHP-induced intracellular reactive oxygen species (ROS) accumulation. In parallel, the activity of anti-oxidant response element (ARE) promoter construct (pARE-Luc), being transfected in cultured astrocytes, was markedly induced by application of ATR oil and asarones. The mRNAs encoding anti-oxidant enzymes, e.g. glutathione S-transferase (GST), glutamate-cysteine ligase modulatory subunit (GCLM), glutamate-cysteine ligase catalytic subunit (GCLC) and NAD(P)H quinone oxidoreductase (NQO1) were induced by ATR oil and asarones in a dose-dependent manner. The ATR oil/asarone-induced gene expression could be mediated by Akt phosphorylation; because the applied LY294002, a phosphoinositide 3-kinase inhibitor, fully abolished the induction. These results demonstrated that α-asarone and β-asarone could account, at least partly, the function of ATR being a Chinese medicinal herb.

## Introduction

The pathogenesis of neurological disorders is still not clearly identified, and oxidative stress in neuronal cell has been proposed to play roles in disease processes [1–2]. The oxidative stress status of cell and tissue could be altered by exposure to oxidants. Reactive oxygen species (ROS) is generated under oxidative stress, and the excess generation of ROS can damage cells [3]. Several lines of evidence revealed that Akt phosphorylation activated nuclear factor-erythroid 2-related factor (Nrf2) and therefore which controlled the expressions of various anti-oxidant and detoxification enzymes via Nrf2-ARE pathway [4]. The expressions of most anti-oxidant enzymes are regulated by transcriptional factor Nrf2 and anti-oxidant response element (ARE). The ARE-driven genes include glutathione S-transferase (GST), glutamate-cysteine ligase modulatory subunit (GCLM), glutamate-cysteine ligase catalytic subunit (GCLC) and NAD(P)H quinone oxidoreductase (NQO1). Glutathione (GSH) is an anti-oxidant preventing the damage by ROS to cellular components [5]. GSH is synthesized by the consecutive action of two enzymes, GCLC and GCLM. Moreover, GST and NQO1 play important role in the detoxification of ROS [6].

Acori Tatarinowii Rhizoma (ATR, the dried rhizome of *Acorus tatarinowii* Schott) has long been one of the most important traditional herbal medicines in Asian countries for at least 2,000 years. The major active components of ATR are α-asarone and β-asarone [7]. Several lines of evidence have suggested the application of ATR and its main ingredients, i.e. α-asarone and β-asarone, in treatment of neurological disorders, especially in neuroprotection [8–11]. Due to these clinical and bioactive properties, the neuroprotective function of α-asarone, β-asarone and volatile oil from ATR were studied. Here, the neuroprotective activities of α-asarone, β-asarone and ATR oil were determined in tert-butyl hydroperoxide (tBHP)-induced rat primary astrocytes. The α- asarone, β-asarone and ATR oil exhibited promising protective effect on the cultures. In addition, the expression of ARE-mediated genes, and the activation of Akt phosphorylation were induced by the asarones in cultured rat astrocytes.

## Materials and methods

### Plant materials, reagents and chemicals

α-Asarone (>98%) and β-asarone (>98%) were kindly provided by Testing Laboratory for Chinese Medicine (Hong Kong, China). Ultra-pure water was prepared from a Milli-Q purification system (Millipore, Molsheim, France). ATR, the dried rhizome of *A*. *tatarinowii*, was purchased from herbal market in Hong Kong. The authentication of plant materials was performed by Dr. Tina T. X. Dong, according to their morphological characteristics stipulated in Chinese Pharmacopeia (2015). The corresponding voucher specimens for ATR (ATR-1-2014) were deposited in Center for Chinese Medicine R&D at Hong Kong University of Science and Technology. LY294002, tert-butyl hydroperoxide (tBHP), tert-butylhydroquinone (tBHQ), 3-(4, 5-dimethylthiazol-2-yl)-2, 5-diphenyl tetrazolium bromide (MTT) (> 98%) were purchased from Sigma Aldrich (St. Louis, MO). The total volatile oil extraction was according to the method stated in Chinese Pharmacopeia (2015). ATR herb was minced and soaked in water in a proportion of 1:8 (w/v) for overnight. The mixture was submitted to hydro-distillation in a Clevenger-type apparatus for 4 hours. Volatile oil was dried over anhydrous sodium sulfate and stored at -20°C. The extraction efficiency was over 95%.

### Cell cultures

Sixty postnatal Sprague-Dawley (SD) rats were obtained from The Animal and Plant Care Facility of HKUST. The experimental procedures had been reviewed and approved by Animal Ethics Committee at HKUST. Primary cultured astrocytes from postnatal day 1 SD rats were isolated and cultured with minor modifications [12]. Cells were maintained in Dulbecco's Modified Eagle Medium (DMEM) with 10% fetal bovine serum (FBS), supplemented with 100 U/mL penicillin and 100 μg/mL streptomycin in a humidified 5% CO_2_ at 37°C. Fresh medium was supplied every 3 days. Culture medium was changed into DMEM supplemented with 0.5% FBS, 100 U/mL penicillin and 100 μg/mL streptomycin 3 hours before drug treatments. Fresh medium was supplied every other day. Reagents for cell cultures were purchased from Life Technologies (Carlsbad, CA).

### Cell viability and ROS formation assay

Primary cultured rat astrocytes were plated in a 96-well plate and pre-treated with various drugs for 48 hours. Then, the cultures were treated with tBHP for 3 hours. Cell viability test was performed with the addition of thiazoly blue tetrazolium bromide (MTT) in PBS buffer at a final concentration of 0.5 mg/mL for 3 hours. The purple precipitate inside the cells was re-suspended in DMSO and then measured at 570 nm absorbance after the removal of solution. The formation of ROS was calculated by using an oxidation-sensitive dye 2’, 7’-dichlorofluorescin diacetate (DCFH-DA) with minor modifications [13]. Cultured rat astrocytes in 96-well plate were pre-treated with α-asarone, β-asarone or ATR oil for 48 hours, then the cells were labeled with 100 μM DCFH-DA in HEPES buffer saline (HBSS) for 1 hour at room temperature in dark. The non-fluorescent dye inside the cells was then hydrolyzed by intracellular esterases to 2’, 7’-dichlorofluorescein (DCFH), and being trapped inside the cells. After washing, cells were treated with tBHP in different concentration for 1 hour. DCFH reacted with various ROS, e.g. hydroxyl radicals and super oxide anions, to form the fluorescent DCF. The signal of intracellular tBHP-induced ROS formation was detected and quantified by EnVision^™^ Multi-label Reader with excitation at 485 nm and emission at 530 nm (Perkin-Elmer, Waltham MA).

### Total anti-oxidant capacity assay

The total anti-oxidant capacity (TAC) was measured by a commercially available assay kit according to the manufacturer's instructions (Biovison, Milpitas, CA). This assay aimed to analysis the anti-oxidants by preventing Cu^2+^ reduction by protein, and which allowed only Cu^2+^ ion to be converted to Cu^+^. The reduced Cu^+^ ion was chelated with a colorimetric probe giving a broad absorbance peak at 570 nm, proportional to the total anti-oxidant capacity. The reaction was started directly, when 100 μL was added immediately with 100 μL of Cu^2+^ working solution in the plate. Plates were covered and incubated at room temperature for 1.5 hours. Trolox, a water-soluble tocopherol analogue, was used as a reference standard. Standard curves were obtained by using increasing concentrations of Trolox in same volume as for the sample. The TAC value of the samples tested was expressed as an equivalent of μM concentration of Trolox solution.

### DNA transfection and luciferase activity

To study the transcriptional activation of anti-oxidant enzymes, pARE-Luc (Promega, Fitchburg, WI) DNA construct was applied. The vector, pGL4.37 [luc2P/ARE/Hygro], contains four copies of an anti-oxidant response element (ARE; 5’-TAG CTT GGA AAT GAC ATT GCT AAT GGT GAC AAA GCA ACT TT-3’) that drives transcription of luciferase reporter gene luc2P (*Photinus pyralis*), when Nrf2 binds to ARE for further gene activation. Cultured rat astrocytes, a well-known study model in analyzing the neuroprotective effect against oxidation and other insults, were transfected with pARE-Luc by Lipofectamine 3000 (Invitrogen, Carlsbad, CA) according to the manufacturer's instructions. The transfection efficiency in rat astrocytes was 30%, as determined by a control plasmid of having β-galactosidase under a cytomergalovirus enhancer promoter. The pARE-Luc transfected astrocytes, cultured in 24-well plates, were treated with various drugs for 48 hours. The medium was aspirated, and the cultures were washed by ice-cold PBS for twice. The cells were lysed by a buffer containing 0.2% Triton X-100, 1 mM dithiothreitol and 100 mM potassium phosphate buffer (pH 7.8) at 4°C. The supernatant was collected and conducted the luciferase assay followed by centrifugation at 13,200 rpm for 10 min at 4°C (Tropix Inc., Bedford, MA). The luminescent reaction was quantified in a GlomaxTM 96 microplate luminometer, and the activity was expressed as absorbance (up to 560 nm) per mg of protein [14].

### Real time PCR analysis

Primary cultured rat astrocytes were treated with α-asarone, β-asarone or ATR oil for 48 hours. Total RNAs were isolated by TRIzol reagent (Invitrogen), and 3 μg of total RNA was reverse-transcribed by Moloney Murine Leukemia Virus (MMLV) reverse transcriptase according to the manufacturer's instruction (Invitrogen). The expression of detoxifying enzymes was determined by quantitative PCR. The primers were as follows; GCLM forward: 5’-CCT GCT GTG TGA TGC CAC CAG ATT TT-3’ and reverse: 5’- TCT GCT TTT CAC GAT GAC CGA GTA CC-3’ (197 bp; NM_017305.2); GCLC forward: 5’- CGT GGA CAC CCG ATG CAG TAT TCT G-3’ and reverse: 5’-GGG TCG CTT TTA CCT CCA CTG TAC T-3’ (261 bp; NM_012815.2); GST forward: 5’-CCT GGG CAT CTG AAA CCT TTT GAG AC-3’ and reverse: 5’-GCG AGC CAC ATA GGC AGA GAG C-3’ (180 bp; L29427); NQO1 forward: 5’-GAC CTT GCT TTC CAT CAC CAC CGG-3’ and reverse: 5’-GTA GAG TGG TGA CTC CTC CCA GAC-3’ (241 bp; NM_017000.3). The 18S rRNA was used as an internal control in all cases and its sequence were as forward: 5’-TGT GAT GCC CTT AGA TGT CC-3’ and reverse: 5’-GAT AGT CAA GTT CGA CCG TC-3’ (320 bp; NR_003286). The PCR condition was set as 94°C (1 min), 60°C (1 min) and 72°C (1 min) for 40 cycles. The real-time PCR was performed by using Fast Start Universal SYBR Green Master (ROX) according to manufacturer's instructions (Applied Bioscience, Foster city, CA). SYBR green signal was detected by ABI 7500 Fast Real-Time PCR system (Applied Biosystems, Foster City, CA). Transcript levels were quantified by using ΔΔCt value method. Calculation was done by using Ct value of 18S of normalize Ct value of target gene in each sample to obtain ΔCt value, which then was used to compare among different samples. PCR products were analyzed by melting curve analysis to confirm specific amplifications [15].

### Western blot and phosphorylation analysis

Cultured rat astrocytes were seeded onto a 12-well plate. The cultures were serum-starved with or without an Akt inhibitor LY294002 for 3 hours before the drug applications. After drug treatments at different time points (0,5,10 and 30 min), the cultures were collected immediately in 2X direct lysis buffer (125 mM Tris-HCI, 2% SDS, 10% glycerol, 200 mM 2-mercaptoethanol, pH 6.8), and the proteins were subjected to SDS-PAGE analysis. Proteins were separated by an 8% SDS-polyacrylamide gels and transferred to the nitrocellulose membrane. Transfer and equal loading of samples was confirmed by Ponceau S staining. The nitrocellulose membrane was blocked with 5% fat-free milk in TBS-T (20 mM Tris base, 137 mM NaCl, 0.1% Tween-20, pH 7.6) for 2 hours at room temperature. Phosphorylated Akt was recognized by anti-phospho-Akt antibody (1:2,500; Cell Signaling, Danvers, MA) at 4°C for overnight, and horseradish peroxidase (HRP)-conjugated anti-rabbit secondary antibody (1:2,500; Invitrogen) for 2 hours at room temperature. After intensive washing with TBS-T, the immune complexes were visualized by using the enhanced chemiluminescence (ECL) method (GE Healthcare, Piscataway, NJ). The intensities of bands in the control and different samples running on the same gel and under strictly standardized ECL condition, were compared on an image analyzer, using a calibration plot constructed from a parallel gel with serial dilutions of one of the samples [16].

### Other assays

A light microscope (Carl Zeiss, Jena, Germany) equipped with a phase-contrast condenser, 10X objective lens, and a digital camera (Carl Zeiss) was used to capture the image with manual setting. The protein concentrations were measured by Bradford's method with a Bio-Rad Laboratories kit (Hercules, CA). Data were expressed as the mean ± standard error of the mean (SEM) for *n* = 3–5. Statistical tests were done by using one-way analysis by Prism 5.00. Differences from basal or control values (as shown in the plots) were classed as significant (*) where *p* >0.05, (**) where *p* >0.01, (***) where *p* >0.001.

## Results

### Anti-oxidant properties of α-asarone, β-asarone and ATR oil

**Fig 1:**
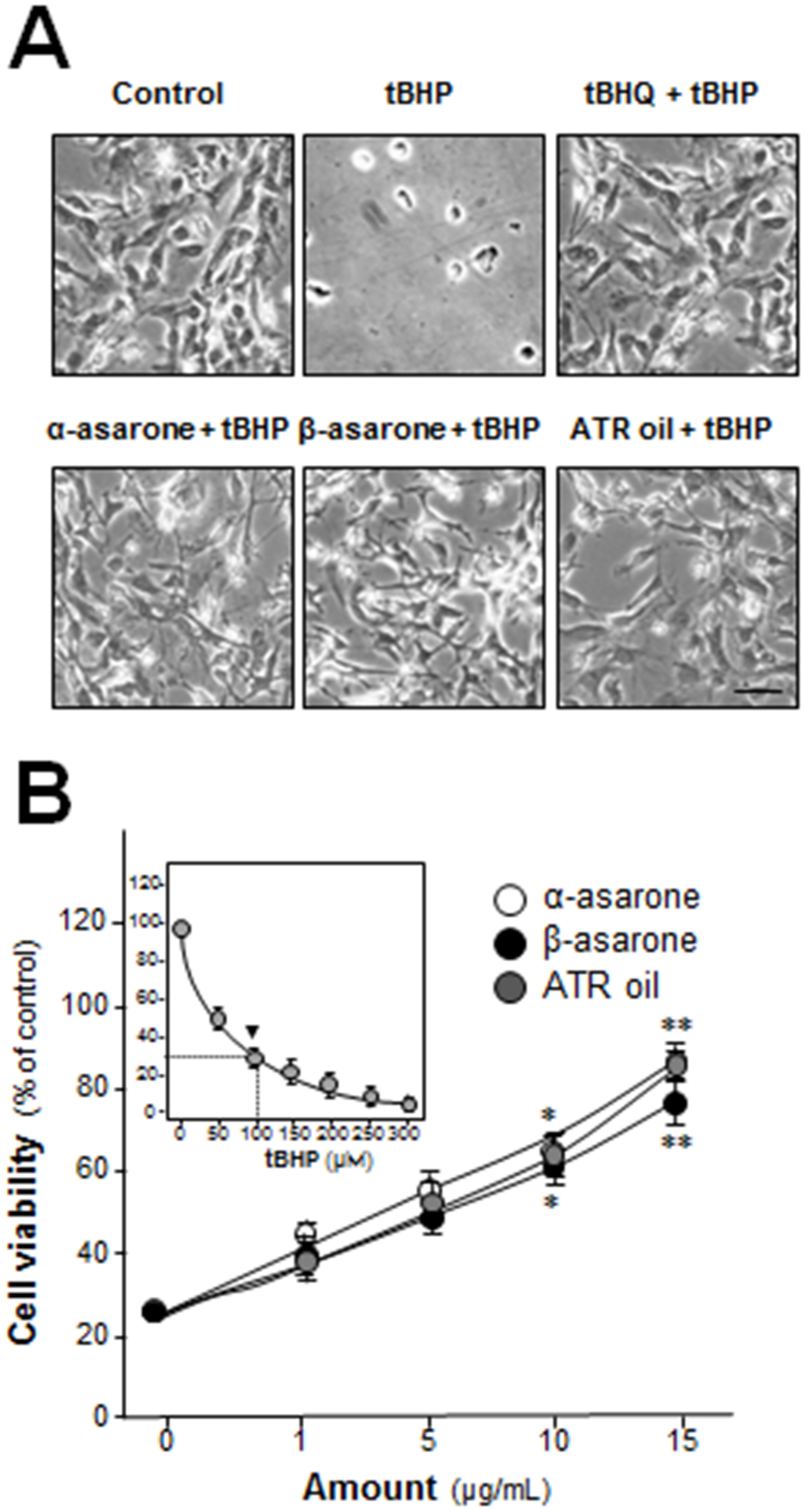
α-asarone, β-asarone or ATR oil protects tBHP-induced cytotoxicity in cultured astrocytes. (A) Morphological changes of tBHP-treated astrocytes were shown. Astrocytes were pre-treated with medium, α-asarone, β-asarone or ATR oil (both at 15 μg/mL) and tBHQ (1.5 μM) for 48 hours before the addition of tBHP (100 μM) for 3 hours. Then cultures were washed with PBS and fixed with 4% paraformaldehyde. Bar = 100 μm. Representative views were shown. (B) Astrocytes (2 × 10^4^ cells/well) were exposed to tBHP at various concentrations (0–300 μM) for 3 hours. Cell viability was expressed as % of control (cells without tBHP) (insert). One hundred μM tBHP (marked) having cell death of ~30% was chosen for subsequent studies. α-Asarone, β-asarone or ATR oil (both at 1–15 μg/mL) was pre-treated the cells for 48 hours before addition of tBHP (100 μM) for cytotoxicity test. Cell viability was detected by MTT assay. Data are expressed as mean ± SEM, where *n* = 5, each with triplicate samples. *p < 0.05; **p < 0.01 compared with control.

α-Asarone and β-asarone were considered as major ingredients of ATR (S1 Fig). Here, the cyto-protective effects of α-asarone, β-asarone or ATR oil against oxidative stress-induced cell injury in cultured astrocytes were analyzed. The morphological changes of tBHP-treated cultured astrocytes were revealed under light microscope. The ATR oil/asarone-treated cultures remained healthy, even in the present of tBHP (Fig 1A). The application of tBHP in the cultures induced oxidative stress and resulted in cell death: the cell viability was significantly decreased by tBHP application in a dose-dependent manner (Fig 1B **insert**). The application of α-asarone, β-asarone or ATR oil protected cell death induced by tBHP challenge. The tBHP-induced cell mortality in cultured astrocytes was markedly reduced by the pre-treatment of α-asarone, β-asarone or ATR oil in a dose-dependent manner (Fig 1B). All concentrations (0.5 to 15 μg/mL) of applied α-asarone, β-asarone or ATR oil, did not show cytotoxicity nor proliferating effect on the cultures (S2 Fig).

**Fig 2:**
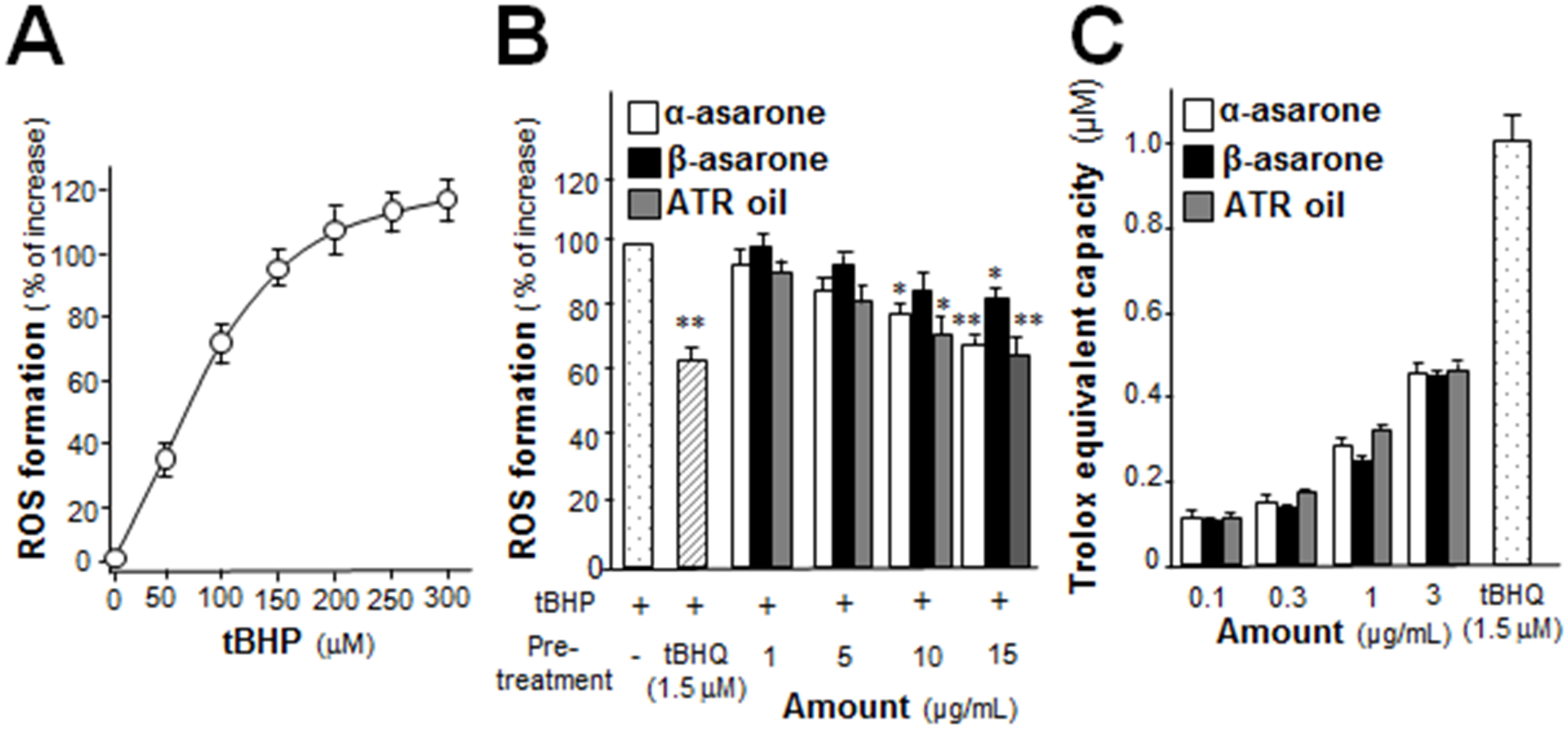
α-asarone, β-asarone or ATR oil suppresses tBHP-induced ROS formation and total anti-oxidant capacity. (A) Cultured astrocytes were exposed to tBHP (0–300 μM) for 1 hour. The level of intracellular ROS was measured. (B) Cultured astrocytes were pre-treated with α-asarone, β-asarone or ATR oil (both at 1–15 μg/mL) and then exposed to tBHP (100 μM) for 1 hour. tBHQ (1.5 μM) was used as positive control of having 40% of ROS inhibition. The results were in % of increase against ROS formation relative to the control (with tBHP alone). (C) Total anti-oxidant capacity of α-asarone, β-asarone and ATR oil at varying concentration were calculated and compared with Trolox equivalent expressed as μM. Data were expressed as mean ± SEM, *n* = 3–5, each with triplicate samples. *p < 0.05; **p < 0.01 compared with control.

The generation of ROS by tBHP was evaluated using dichlorofluorescein (DCF) fluorescence assay [17]. In cultured astrocytes, an 1-hour treatment of tBHP could increase intracellular ROS formation in a dose-dependent manner (Fig 2A). The tBHP-induced ROS formation was reduced by 40% after pre-treatment of tBHQ, a known anti-oxidant (Fig 2B). The pre-treatment of α-asarone, β-asarone or ATR oil reduced ROS level in a dose-dependent manner (Fig 2B). Under different pathophysiological conditions, e.g. heart and vascular diseases, diabetes mellitus, renal disorders and neurological disorders, total anti-oxidant capacity could be a reliable biomarker of diagnostics and prognostics. The total anti-oxidant capacity of α-asarone, β-asarone or ATR oil was measured here, and which were compared in different concentrations, as shown in Fig 2C.

### Asarone or ATR oil induces anti-oxidant enzymes

**Fig 3:**
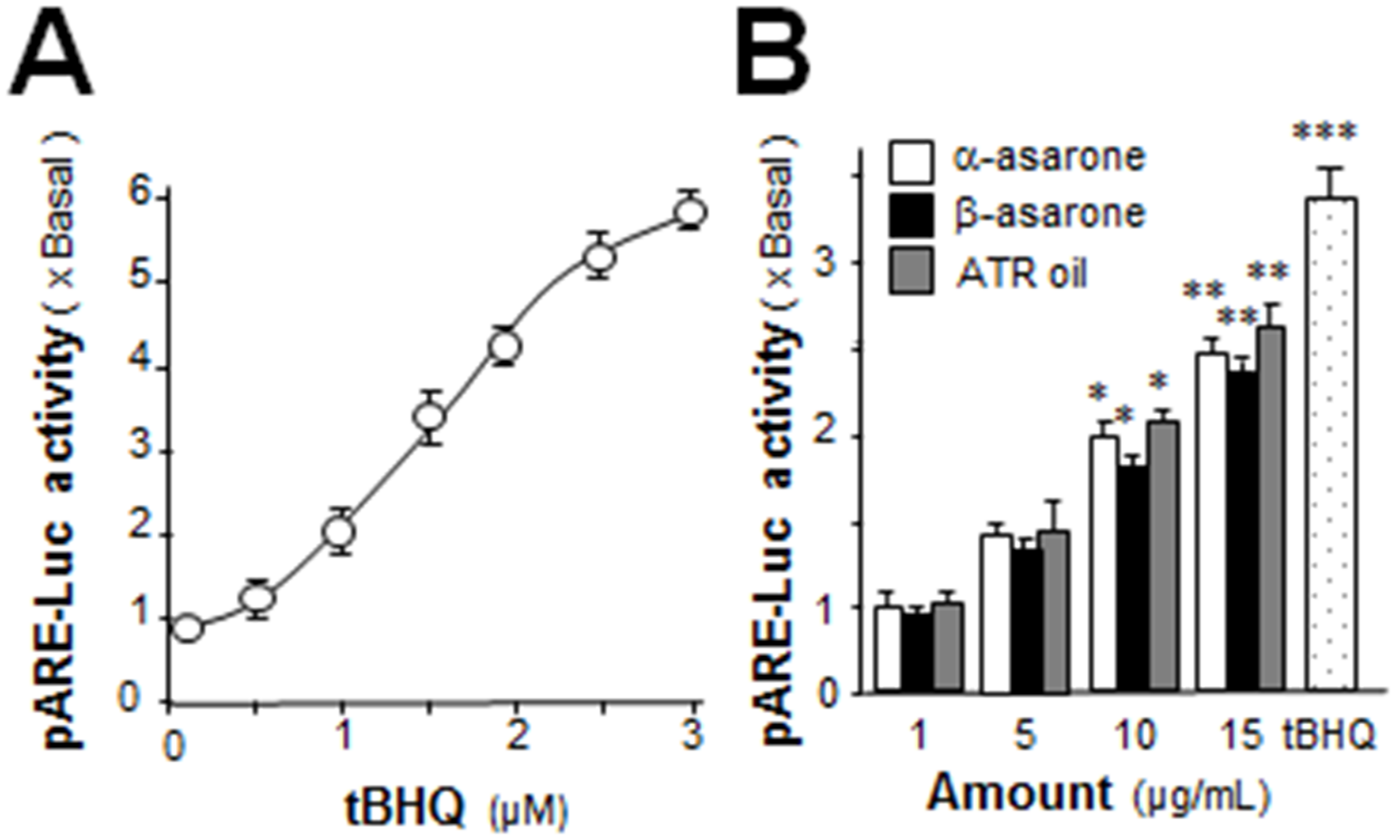
α-asarone, β-asarone or ATR oil induces ARE transcriptional activity in cultured astrocytes. (A) Cultured astrocytes, transfected with pARE-Luc, were treated with tBHQ (0–2.5 μM) for 48 hours. The cell lysates were subjected to luciferase assay to measure the activity driven by ARE. (B) α-Asarone, β-asarone or ATR oil (both at 1–15 μg/mL) was applied onto pARE-Luc-expressed astrocytes for 48 hours. The cell lysates were subjected to luciferase assay. tBHQ (1.5 μM) was used as a positive control. Data are expressed as the fold of increase to basal reading (untreated culture), and they are in mean ± SEM, where *n* = 4, each with triplicate samples. *p < 0.05; **p < 0.01; ***p < 0.001 compared with control.

To study the role of α-asarone, β-asarone or ATR oil in transcriptional regulation of ARE reporter gene, cultured astrocytes were stably transfected with a promoter-reporter construct containing four repeats of ARE tagged with a luciferase reporter gene (pARE-Luc), and which were treated with tBHQ, α-asarone, β-asarone or ATR oil. After the treatment, the cells were lysed and subjected to luciferase measurement. tBHQ, served as a positive control, induced the promoter activity in a dose-dependent manner (Fig 3A). As shown in Fig 3B, α-asarone, β-asarone and ATR oil induced ARE transcriptional activity in a dose-dependent manner. The maximal induction of luciferase activity was at ~2.5 folds at 15 μg/mL asarones or ATR oil.

**Fig 4:**
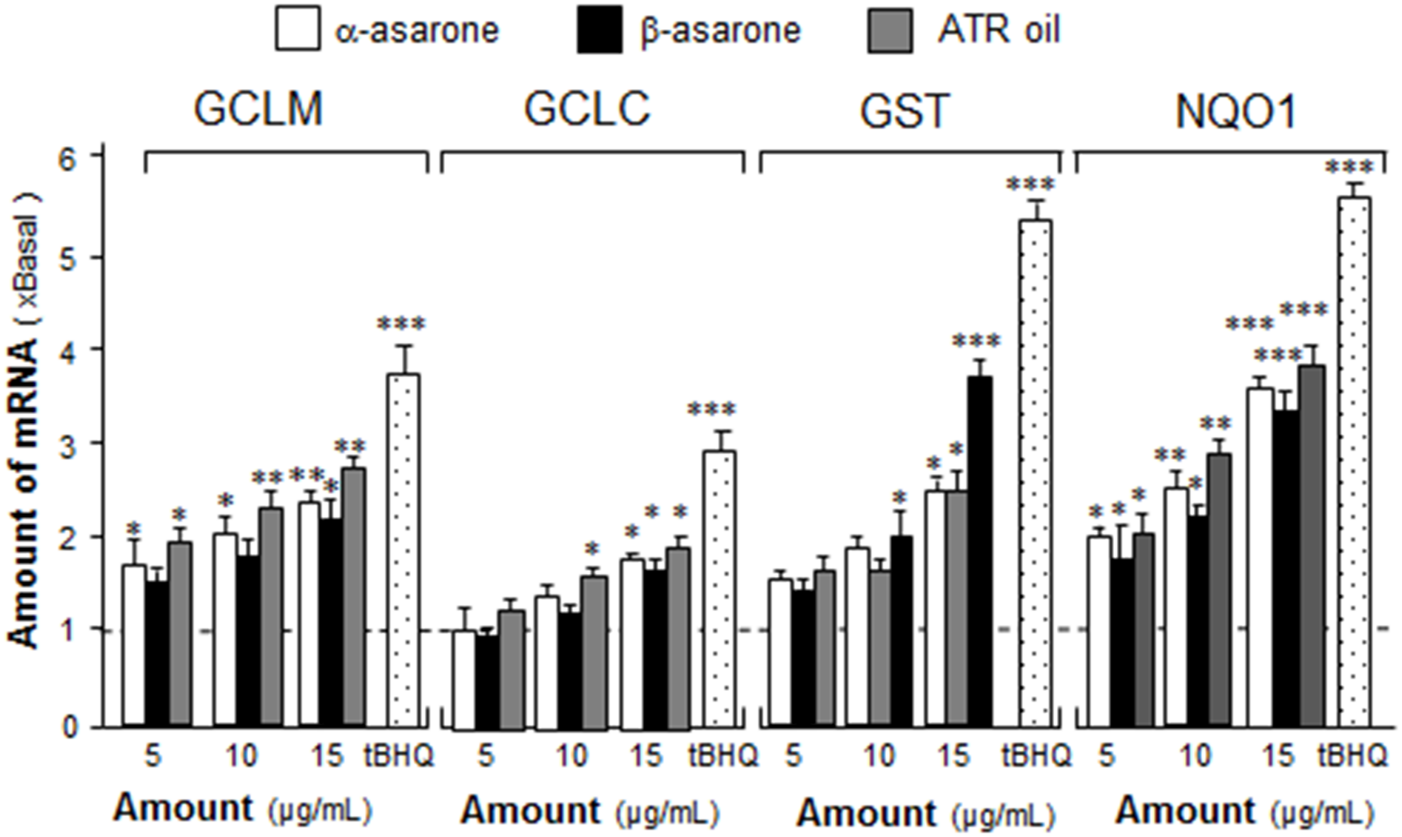
α-asarone, β-asarone or ATR oil induces the expression of anti-oxidant enzymes in cultured astrocytes. Cultured astrocytes were treated with α-asarone, β-asarone or ATR oil (both at 5–15 μg/mL) or tBHQ (1.5 μM) for 48 hours. Total RNAs were isolated from cultured astrocytes and then reversed transcribed into cDNAs for the detection of mRNAs encoding GCLC, GCLM, NQO1 and GST by real-time PCR analysis. The 18S served as internal control. Values are expressed as the fold of increase to basal reading (untreated culture), and in mean ± SEM, where *n* = 3, each with triplicate samples. *p < 0.05; **p < 0.01 compared with control.

ARE is a specific enhancer inducing the gene expressions of many phase ΙΙ detoxifying enzymes [18]. The detoxifying enzymes are vital biomarkers of neuroprotection: they are GST for detoxification of xenobiotic and electrophiles, GCLC and GCLM for glutathione (GSH) biosynthesis, and NQO1 for protecting against deleterious reactive semi-quinones by converting exogenous quinones into hydroquinones. According to the results of pARE-Luc activation, the roles of α-asarone, β-asarone and ATR oil in expression of detoxifying enzymes were determined in cultured astrocytes by real-time qPCR. To reveal the tropic effect of asarone, cultured astrocytes were treated with α-asarone, β-asarone or ATR oil for 48 hours, total RNA was collected and subjected to real time quantitative PCR analysis by using specific primers flanking NQO1, GCLC GCLM and GST mRNAs. α-Asarone, β-asarone and ATR oil stimulated the expressions of NQO1, GCLC, GCLM and GST by 2 to 3 folds, when compared to the control (Fig 4).

### Asarone or ATR oil activates Akt signaling pathway

**Fig 5:**
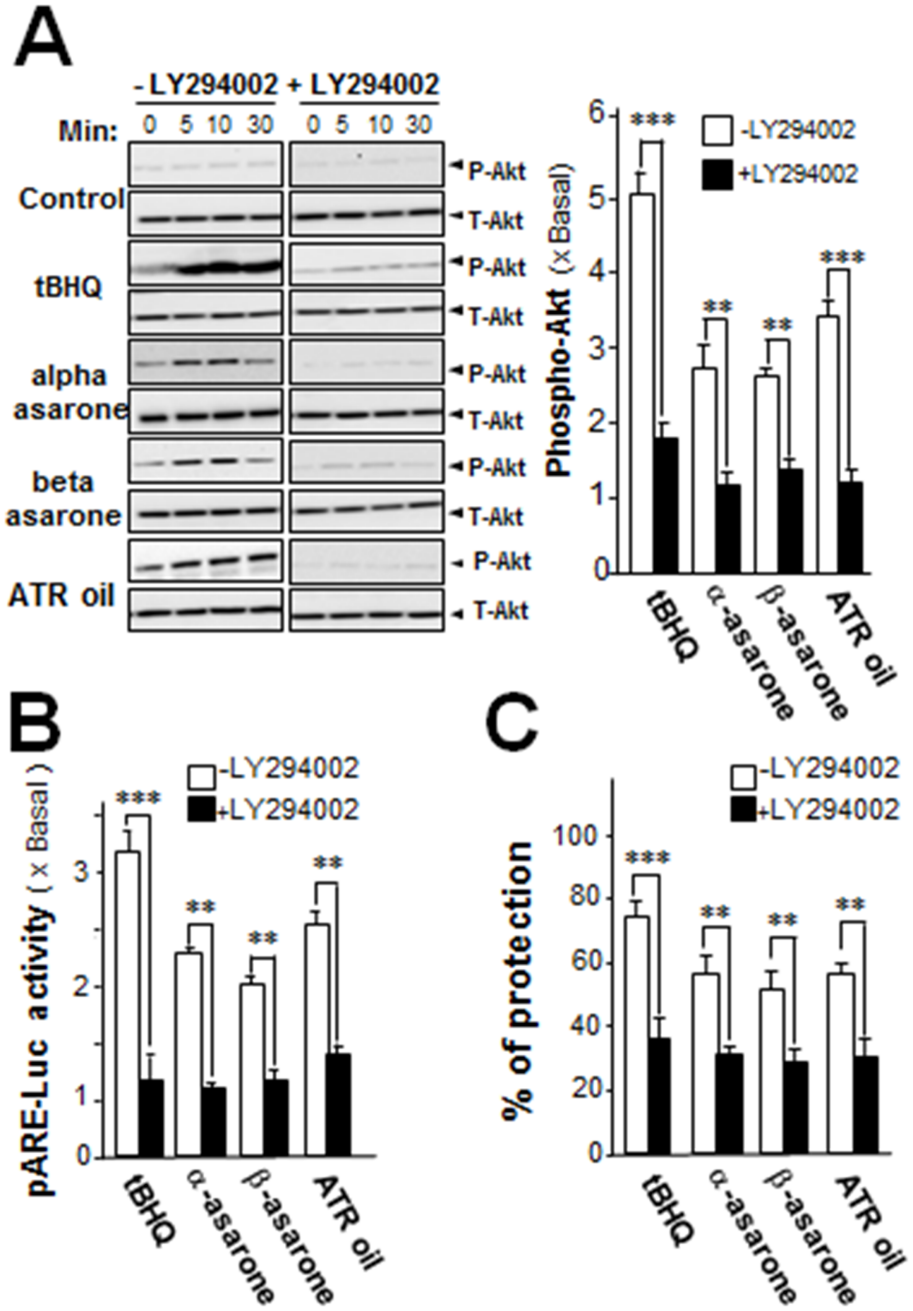
α-asarone, β-asarone or ATR oil activates the phosphorylation of Akt in cultured astrocytes. (A) Serum free astrocytes pre-treated with DMEM or LY294002 (10 μM) for 3 hours prior to treatment of α-asarone, β-asarone or ATR oil (both at 15 μg/mL) for indicated time (5, 10 and 30 min). Phosphorylation of Akt was detected by immune blot analysis using specific antibodies. Band density was estimated, densitometrically, and the phosphorylation rates were expressed as the intensity of phosphorylated Akt relative to total Akt (p-Akt/t-Akt). (B) Cultures, transfected with pARE-Luc, were pre-treated with medium or LY294002 (10 μM) for 3 hours prior to treatment of 15 μg/mL α-asarone, β-asarone or ATR oil for 48 hours. (C) Cultures were pre-treated with medium or LY294002 as in (B) for 48 hours. Then, the cells were challenged with tBHP (100 μM) for 3 hours. The cell viability was determined. Values are in mean ± SEM, where *n* = 3, each with triplicate samples. *p < 0.05; **p < 0.01 compared with control.

Activation of Akt signaling has been shown to be important for neuroprotection [19–20]. The impact of α-asarone, β-asarone or ATR oil on activation of Akt was studied. Cultured astrocytes were treated with α-asarone, β-asarone or ATR oil for various time periods, and the cell lysates were subjected to western blot analysis with anti-phospho-Akt-specific antibody. α-Asarone, β-asarone and ATR oil dose-dependently activated phosphorylation of Akt at ~60 kDa, the induction of Akt phosphorylation at 3 folds was revealed after 10 min of treatment (Fig 5A **left panel**). In line with luciferase results and protection against the oxidative stress-induced cell injury, the pre-treatment of a phosphoinositide 3-kinase (PI3K) inhibitor, LY294002, blocked the ATR oil/asarone-mediated phosphorylation (Fig 5A **right panel**). These results supported the activation of Akt signaling by asarone and ATR oil was an essential step in protecting astrocytes from oxidative stress-induced cell injury.

To further investigate the role of Akt in ARE transcriptional activation, astrocytes were pre-treated with LY294002, before the treatment of asarone or ATR oil. The ARE-driven luciferase activity was significantly abolished by pre-treatment of inhibitor, as compared with the control (Fig 5B**)**. The results suggested that the ATR oil/asarone -induced ARE transcriptional activation was mediated by Akt activation. To determine the role of Akt in ATR oil/asarone-mediated cyto-protection against tBHP-induced cell injury, cultured astrocytes were pre-treated with LY294002 for 3 hours, and then the cells were treated with asarone or ATR oil for 48 hours. The application of LY294002 exerted cytotoxicity on tBHP-treated cells. The protective effect of asarone or ATR oil was significantly reduced in presence of LY294002 (Fig 5C). This result supported the notion that activation of Akt signaling by α-asarone, β-asarone or ATR oil was a key step in protecting cells from oxidative stress-induced cell injury.

## Discussion

World Health Organization reports that millions of people around the world suffer from various types of neurological disorders, e.g. Alzheimer and Parkinson diseases, strokes, multiple sclerosis, brain injuries and neuro-infections. Oxidative stress can be viewed as an imbalance of defense mechanism of anti-oxidants and over production or incorporation of free radicals from outside environment to living system, which results in serious penalty leading to neuro-degeneration [21]. Finding ways to reduce the mortality of neurological disorders remain a vital public health goal. Here, we identified asarone, an ingredient deriving from a Chinese medicinal herb ATR, could be one of the agents in preventing the onset of neurological disorders. In terms of chemical composition, α-asarone and β-asarone (cis- and trans-form of asarone) are the major chemicals (more than 95%) in essential oil of ATR [22–23], and historically ATR essential oil has a wide range of usages in herbal decoctions and beverages. Our previous studies showed that the natural ratio of α-asarone and β-asarone in ATR oil is about 1:4. Furthermore, α-asarone and β-asarone together showed synergistic effects in this ratio in potentiating the NGF-induced neuronal differentiation in cultured PC12 cells [7]. Although ATR is general considered as a safe Chinese herb, the exploration of ATR clinical applications has drawn our attention on its toxic characteristics of β-asarone, which is more toxic than that of α-asarone. The Council of the European Communities has stated that the permissible level of β-asarone (0.1 mg/kg in food products and beverages), higher dosage of intake should be restricted. Caution is required for the dosage regimens of asarones; only safe and effective dose of asarones could be studied for their promising protective effect. The dose selection and treatment duration should be given appropriately in order to avoid toxicity.

α-Asarone and β-asarone (3, 10, 30 mg/kg, i.p.) have anti-oxidant properties in various animal seizure models [24–25]: these compounds play vital protective role in normalizing increase of superoxide dismutase and lipid peroxidation, as well as decrease of catalase and glutathio-peroxidase, in different regions of rat brain under stressed condition. But the anti-oxidative property and action mechanism of α-asarone and β-asarone in neuronal cells are still not clear. Here, α-asarone, β-asarone and ATR oil were suggested to exhibit neuroprotective activities in several assays.

The redox-sensitive transcription factor Nrf2 plays a critical role in protecting cells against oxidative stress, which binds ARE sites leading to up-regulation of detoxifying enzymes [26]. α-Asarone, β-asarone and ATR oil markedly suppressed ROS formation during oxidative injury, therefore the effects of α-asarone, β-asarone and ATR oil on Nrf2-dependent ARE-driven genes, including NQO1, GCL and GST, were determined. These ARE-driven genes have been demonstrated to play an important role in protecting cells against oxidative stress [27]. NQO1 is a flavor-enzyme catalyzing the two-electron reduction of quinines. Consequently, it prevents the electron reduction of quinone that results in production of radical species. GCL consists of catalytic (GCLC) and modifier (GCLM) subunits, which is the rate-limiting enzyme mediating the biosynthesis of glutathione (GSH). GSH, a tripeptide, is an anti-oxidant, which could prevent damage to cellular components caused by ROS. Here, a marked induction of GCLC and GCLM were observed in astrocytes after treatment with asarone or ATR oil. Thus, these data suggested that an induction of GCL might be involved in the asarone/ATR oil-mediated elevation of cellular GSH.

The Akt-dependent protein kinase is one of the upstream activators of Nrf2. And the regulation of detoxifying enzymes has been focusing on the role of Akt [7]. Our results showed that α-asarone, β-asarone and ATR volatile oil up regulated Akt phosphorylation as well as ARE transcriptional activation in cultured astrocytes. The ATR oil/asarone-induced Akt activation could be followed the well-known signaling of phos- phoinositide 3-kinase (PI3K). The translocation of Akt from cytoplasm to plasma membrane depends on the pleckstrin homology (PH) domain. Besides, phosphoinositide-dependent kinase 1 (PDK1) contains a PH domain that binds directly to PIP2 and PIP3, triggering its translocation to plasma membrane upon activation of PI3K. The co-localization of activated PDK1 and Akt was leading to the phosphorylation of Akt, which could be prevented by a PI3-kinase inhibitor LY294002 [28–29]. These results indicated that the neuroprotective effect of α-asarone, β-asarone and ATR oil against oxidative stress-induced cell death in astrocytes could be triggered by: (i) protecting tBHP-induced cytotoxicity; (ii) decreasing intracellular ROS production; (iii) inducing ARE transcriptional activity; (iv) inducing anti-oxidant enzymes; and (v) activating Akt phosphorylation.

## Conclusion

Our study focused on the neuroprotective role of α-asarone, β-asarone or ATR oil in cultured rat astrocytes. The treatment of α-asarone, β-asarone or ATR oil protected cultured rat astrocytes from oxidative stress, which was shown to be mediated by scavenging of ROS formation and stimulating the Nrf2-ARE self-defense mechanism. Furthermore, α-asarone, β-asarone or ATR oil activated Akt signaling and, subsequently, triggered the expressions of anti-oxidant enzymes. In conclusion, the neuroprotective effect of asarone or ATR oil offers an exciting therapeutic approach to a number of neuronal diseases.

## Supporting information

S1 Fig HPLC-DAD chromatogram of marker and ATR extract. The identification of α-asarone and β-asarone in standards was made by DAD detector (257 nm). Representative chromatogram are shown, *n* = 3. The identification of α-asarone and β-asarone in ATR was made by DAD detector (257 nm). Representative chromatograms are shown, *n* = 3.
(TIF)

S2 Fig Effect of α-asarone, β-asarone or ATR oil on the viability of astrocytes. Cultured astrocytes were treated with the different doses (0.5 to 30 μg/mL) of α-asarone, β-asarone or ATR oil, for 48 hours. Cell viability (using the colorimetric MTT assay) was performed. No significant increase in cell viability was observed. Values are in mean ± SEM, *n* = 5, each with triplicate samples.
(TIF)

## Abbreviations

ARE: anti-oxidant response element
ATR: Acorus Tatarinowii Rhizoma
Nrf2: nuclear factor-erythroid 2-related factor
ROS: Reactive oxygen species
TAC: total anti-oxidant capacity
tBHP: tert-butyl hydroperoxide
