# Supplementary figures and images for: Asarone from Acori Tatarinowii Rhizome prevents oxidative stress-induced cell injury in cultured astrocytes: A signaling triggered by Akt activation
Source: PLoS One. 2017 Jun 9;12(6):e0179077. doi: 10.1371/journal.pone.0179077 (PMC5466315; doi:10.1371/journal.pone.0179077)

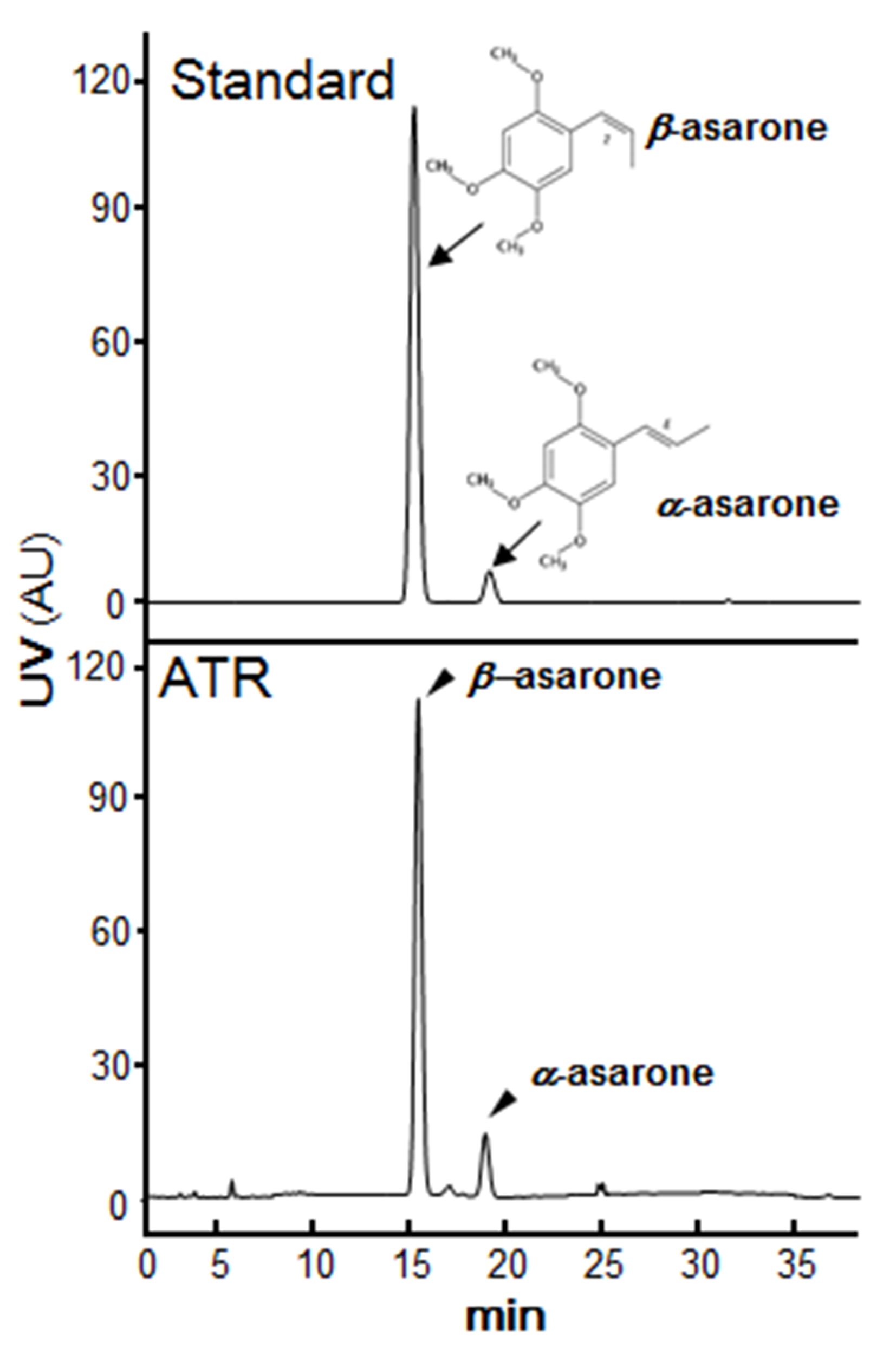

Supplement: S1 Fig — The identification of α-asarone and β-asarone in standards was made by DAD detector (257 nm). Representative chromatogram are shown, n = 3. The identification of α-asarone and β-asarone in ATR was made by DAD detector (257 nm). Representative chromatograms are shown, n = 3. (TIF) [file pone.0179077.s001.tif]

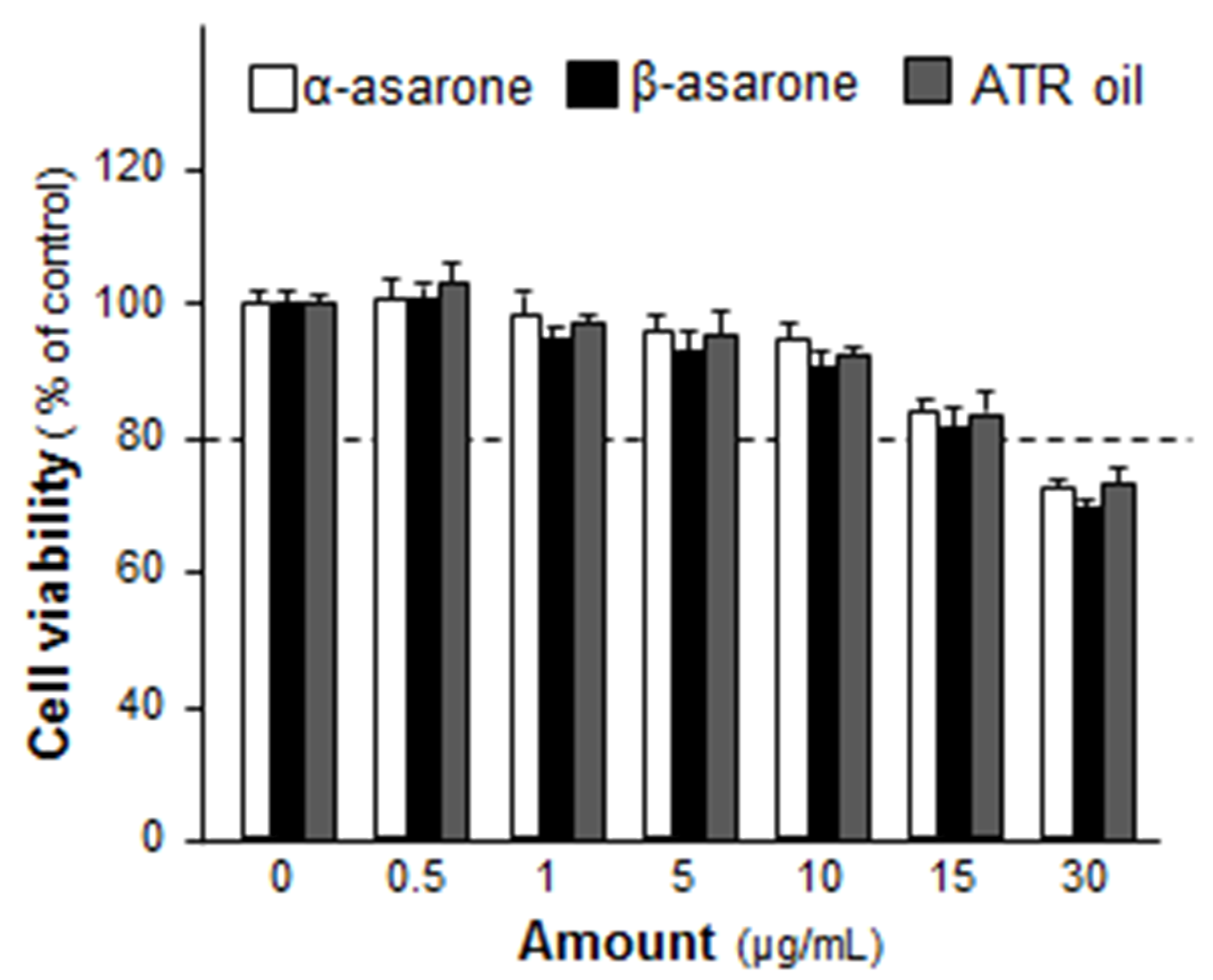

Supplement: S2 Fig — Cultured astrocytes were treated with the different doses (0.5 to 30 μg/mL) of α-asarone, β-asarone or ATR oil, for 48 hours. Cell viability (using the colorimetric MTT assay) was performed. No significant increase in cell viability was observed. Values are in mean ± SEM, n = 5, each with triplicate samples. (TIF) [file pone.0179077.s002.tif]
